# *MLL2/KMT2D* and *MLL3/KMT2C* expression correlates with disease progression and response to imatinib mesylate in chronic myeloid leukemia

**DOI:** 10.1186/s12935-018-0523-1

**Published:** 2018-02-20

**Authors:** Doralina do Amaral Rabello, Vivian D’Afonseca da Silva Ferreira, Maria Gabriela Berzoti-Coelho, Sandra Mara Burin, Cíntia Leticia Magro, Maira da Costa Cacemiro, Belinda Pinto Simões, Felipe Saldanha-Araujo, Fabíola Attié de Castro, Fabio Pittella-Silva

**Affiliations:** 1 Laboratory of Molecular Pathology of Cancer, Faculty of Health Sciences and Medicine, University of Brasilia, Brasília, DF Brazil; 2 Department of Clinical Analysis, Toxicology and Food Sciences, School of Pharmaceutical Sciences of Ribeirão Preto, University of São Paulo, Ribeirão Preto, SP Brazil; 3 Department of Internal Medicine, School of Medicine of Ribeirão Preto, University of São Paulo, Ribeirão Preto, SP Brazil; 4 Laboratory of Molecular Pharmacology, Faculty of Health Sciences, University of Brasilia, Brasília, DF Brazil

**Keywords:** *MLL2/KMT2D*, *MLL3/KMT2C*, Chronic myeloid leukemia, Genetic alterations, Epigenetic, Lysine methyltransferase

## Abstract

**Background:**

Chronic myeloid leukemia (CML) is a clonal myeloproliferative neoplasm whose pathogenesis is linked to the Philadelphia chromosome presence that generates the *BCR*–*ABL*1 fusion oncogene. Tyrosine kinase inhibitors (TKI) such as imatinib mesylate (IM) dramatically improved the treatment efficiency and survival of CML patients by targeting BCR–ABL tyrosine kinase. The disease shows three distinct clinical-laboratory stages: chronic phase, accelerated phase and blast crisis. Although patients in the chronic phase respond well to treatment, patients in the accelerated phase or blast crisis usually show therapy resistance and CML relapse. It is crucial, therefore, to identify biomarkers to predict CML genetic evolution and resistance to TKI therapy, considering not only the effects of genetic aberrations but also the role of epigenetic alterations during the disease. Although dysregulations in epigenetic modulators such as histone methyltrasnferases have already been described for some hematologic malignancies, to date very limited data is available for CML, especially when considering the lysine methyltransferase *MLL2/KMT2D* and *MLL3/KMT2C*.

**Methods:**

Here we investigated the expression profile of both genes in CML patients in different stages of the disease, in patients showing different responses to therapy with IM and in non-neoplastic control samples. Imatinib sensitive and resistant CML cell lines were also used to investigate whether treatment with other tyrosine kinase inhibitors interfered in their expression.

**Results:**

In patients, both methyltransferases were either upregulated or with basal expression level during the chronic phase compared to controls. Interestingly, *MLL3/KMT2C* and specially *MLL2/KMT2D* levels decreased during disease progression correlating with distinct clinical stages. Furthermore, *MLL2/KMT2D* was decreased in patients resistant to IM treatment. A rescue in the expression of both *MLL* genes was observed in KCL22S, a CML cell line sensitive to IM, after treatment with dasatinib or nilotinib which was associated with a higher rate of apoptosis, an enhanced expression of *p21* (*CDKN1A*) and a concomitant decrease in the expression of *CDK2*, *CDK4* and *Cyclin B1 (CCNB1)* in comparison to untreated KCL22S control or IM resistant KCL22R cell line, which suggests involvement of p53 regulated pathway.

**Conclusion:**

Our results established a new association between *MLL2/KMT2D* and *MLL3/KMT2C* genes with CML and suggest that *MLL2/KMT2D* is associated with disease evolution and may be a potential marker to predict the development of therapy resistance.

## Background

Chronic myeloid leukemia (CML), a myeloproliferative neoplasm, is a clonal disorder involving pluripotent stem cells. The pathogenesis of CML comes mainly from the consequences of the Philadelphia (Ph) chromosome, which results from a translocation involving the chromosomes 9 and 22 (t(9;22)(q34;q11)). There are several possible break points, generating different chimeric *BCR*–*ABL1* transcripts. All of the transcripts code for constitutively active tyrosine kinases that lead to cell overgrowth, proliferation and reduced apoptosis. Whether these transcripts are associated with clinical and molecular characteristics is a controversial discussion that still needs to be clarified.

The disease typically evolves through three distinct clinical stages: chronic phase, accelerated phase and blast crisis. Patients diagnosed during the chronic phase, which constitutes the vast majority of patients (85–90%), can benefit from tyrosine kinase inhibitors (TKI) therapy and usually have a good prognosis. Progression of the disease to accelerated and blast phase usually occurs with the development of therapy resistance. The official criteria to diagnose the accelerated phase is not yet defined and includes provisional parameters related to genetic evolution and therapy resistance. Despite all the advances in our understand of CML, with identification of a few mutations associated with disease progression [1] and with different TKIs available for treatment, there is a lack of early and robust markers that can predict genetic evolution and development of therapy resistance.

The number of studies showing epigenetic regulation in CML has been gradually increasing. A recent report revealed an inverse correlation between the expression level of lysine methyltransferases *EHMT1* and *EHMT2* with the type I interferon responsiveness in CML cell lines. This observation led to the use of EHMT1 and EHMT2 specific inhibitors which sensitized several CML cell lines to interferon and imatinib treatments [2]. Another report showed a decreased expression of the methyltransferase *RIZ1(PRDM2)* during CML progression to blast crisis. The loss of *RIZ1* expression blocked apoptosis and differentiation pathways leading to an increase in myeloid blast cell population resulting in CML progression [3]. In addition, another methyltransferase, *PRDM12*, was mapped to the minimal deleted region flanking *ABL* and *BCR* genes in a set of CML patients with unfavorable prognosis, figuring as a strong candidate tumor suppressor gene [4]. Dysregulation in epigenetic modifiers therefore, affect pathways that help the survival and propagation of leukemic cells. Although the involvement of protein methyltransferases in some hematologic malignancies, such as diffuse large B cell lymphoma, follicular lymphoma, acute myeloid leukemia and multiple myeloma is becoming gradually clear, very few information is available for CML [5, 6].

*MLL2/KMT2D* and *MLL3/KMT2C* are members of the *MLL* (mixed lineage leukemia) or *KMT2* family of genes that encode enzymes containing clustered chromatin-binding PHD zinc fingers, FY-rich regions, DNA recognition domains and the catalytic SET domain, responsible for the methylation of H3K4 which is related to active gene transcription. These proteins form large Set1/COMPASS-like complexes that are recruited to enhancer regions by binding to nuclear receptors and DNA-binding transcription factors, such as p53. In this way, cell expression can be regulated according to transcription factors availability, which will depend on several cell signals, and different interactions will lead to context-dependent functions of these complexes [7, 8]. *MLL* genes have been associated to many different types of cancer [7, 9–11], but to date there is no clear information on the relationship of these genes with CML.

In this study, aiming to detect potential markers to predict genetic evolution and development of therapy resistance, we used an exploratory cohort to investigate the expression profile of *MLL2/KMT2D* and *MLL3/KMT2C* genes in CML, in different disease stages, including patients showing different responses to therapy with imatinib mesylate.

## Subjects and methods

### Patient samples and data collection

Forty-six samples from CML patients and twenty from healthy individuals as controls were included in this study. Among the 46 CML patients, 29 were in indolent chronic phase, 8 were in accelerated phase and 9 were in the more aggressive blast phase. The diagnosis of CML patients was based in the unexplained persistent leukocytosis, the presence of Ph-chromosome abnormality detected by routine cytogenetics; or the Ph-related molecular *BCR*–*ABL* oncogene detected by fluorescent in situ hybridization (FISH) or molecular test. CML patients called IM “resistant” or “responsive” (complete cytogenetic remission after 12 months of IM treatment), were defined according to the criteria proposed by the European Leukemia Net (leukemia-net.org). Clinical and demographic characteristics of the CML patients are summarized in Table 1. The control cohort had 9 female and 11 male individuals, with an average age of 46.3 years (range 22–65 years). In addition, samples from patients with other myeloproliferative diseases including polycythemia vera (N = 25), essential thrombocythemia (N = 35) and primary myelofibrosis (N = 20) were also examined. The study was approved by the Ethics Committee of the School of Pharmaceutical Sciences and the Clinical Hospital of the School of Medicine of Ribeirão Preto, University of São Paulo.

**Table 1:** Clinical and demographic characteristics of the CML patients

Characteristics	No. of patients (%)
Total	46 (100%)
Gender	
Female	24 (52.2%)
Male	22 (47.8%)
Age	
Average	45.2 years
Range	18–69 years
Phase	
Chronic	29 (63%)
Accelerated	8 (17.4%)
Blastic	9 (19.6%)
Sokal Index	
A	11 (23.9%)
B	13 (28.3%)
C	19 (41.3%)
N/A	3 (6.5%)
Response to kinase inhibitor therapy	
Sensitive	17 (37%)
Resistant	22 (47.8%)
N/A	7 (15.2%)

*N/A* not available

### Cell culture and inhibitor treatment

A pair of CML cell lines, IM sensitive (KCL22S) and IM resistant (KCL22R), was used to assess *MLL2/KMT2D* and *MLL3/KMT2C* expression as well as expression of p53 regulated genes *p21 (CDKN1A)*, *Cyclin B (CCNB1)*, *CDK2* and *CDK4*, when treated with other tyrosine kinase inhibitors (dasatinib or nilotinib). KCL22 is a lineage established from a female patient with chronic myeloid leukemia in blast phase, expressing the b2-a2 transcript of BCR–ABL. Cells were cultivated with RPMI 1640 (Thermo Fisher Scientific, Massachusetts, USA) supplemented with 10% fetal bovine serum (Crystalgen, NY, USA), 1% l-glutamine (Gibco™, USA) and 1% streptomycin/penicillin (Gibco™, USA) under a humidified atmosphere with 5% CO_2_ at 37 °C. Both cell lines were seeded at a density of 2 × 10^6^ cells/well, with medium RPMI/10% fetal bovine serum containing 10 nM dasatinib, 18 nM of nilotinib or 10 mM of imatinib. The control group did not receive any drug. Twelve hours after treatment, cells were collected and pelleted for total RNA extraction.

### Cell viability and apoptosis

Cell viability was measured by trypan blue exclusion test. A 0.4% trypan blue dye solution was used to determine the percentage of KCL22 R and S viable cells pre- and post-treatment with the tyrosine kinase inhibitors dasatinib, nilotinib or imatinib, as described above. 10 µl of KCL22 R and S suspension cells of each treatment group were resuspended in PBS, mixed in 190 µl of trypan blue solution and counted using a hemacytometer to determine the number of viable and non-viable blue cells. The percentage of viable cells was calculated by using the formula: % viable cells = [1.00 − (Number of blue cells ÷ Number of total cells)] × 100.

Cell apoptosis was quantified by flow cytometry, using the annexin-V/fluorescein isothiocyanate (FITC) technique. Cell lines were cultured for 12 h, as described above, in the presence of tyrosine kinase inhibitors dasatinib, nilotinib or imatinib, and subsequently recovered by centrifugation, washed with annexin buffer (10 mM Hepes, pH 7.4; 150 mM NaCl; 5 mM KCl; 1 mM MgCl2; 1.8 mM CaCl_2_) and incubated in the dark for 20 min with 5 μl annexin-V/FITC. Then, 5 μl propidium iodide (PI) solution (50 μg/ml) was added to each tube and cell content was analyzed by flow cytometry. Five thousand cells were acquired using FACS Canto Flow Cytometer (Becton–Dickinson, New Jersey, USA) and analyzed by dot-plot with Diva 6.0 Software (Becton–Dickinson, New Jersey, USA). The results are given as percentage of apoptotic cells (cells positive for annexin-V FITC and annexin-V FITC plus PI).

### Cells isolation, RNA extraction, cDNA synthesis and qPCR

Peripheral blood samples from CML patients and healthy individuals (controls) were used to isolate mononuclear cells using Ficoll Paque Plus, according to the manufacturer’s instructions (GE Healthcare, Little Chalfont, UK). Total RNA was extracted from each patient sample and cell lines using TRIzol Reagent (Thermo Fisher Scientific, Massachusetts, USA) according to the manufacturer’s protocol. Single-stranded complementary DNA was generated from total RNA with reverse transcriptase and random primers, using the High Capacity cDNA Reverse Transcription Kit (Thermo Fisher Scientific, Massachusetts, USA). Reactions of quantitative PCR (qPCR) from patients samples or cell lines were performed on a StepOnePlus Real-Time PCR System (Thermo Fisher Scientific, Massachusetts, USA) using TaqMan Gene Expression Assays, according to the manufacturer’s instructions (Hs00231606_m1 for *MLL2/KMT2D,* Hs00419011_m1 for *MLL3/KMT2C,* Hs99999903_m1 for *Beta*-*actin/ACTB,* Hs00259126_m1 for *CCNB1/Cyclin B1*, Hs00355782_m1 for *CDKN1A/P21,* Hs01548894_m1 for *CDK2*, Hs00262861_m1 for *CDK4* and Hs_99999905_m1 for *GAPDH*; Thermo Fisher Scientific, Massachusetts, USA). qPCR assays were carried out in duplicate for each sample, in a final volume of 10 µl. Amplification conditions were as follow: 2 min at 50 °C and 10 min at 95 °C on holding stage, and then 40 cycles of 15 s at 95 °C and 1 min at 60 °C. Relative gene expression was calculated using *GAPDH* and/or *BETA*-*ACTIN* as endogenous control genes to normalize sample input. The average of Ct (cycle threshold) values of CML and normal samples were calculated to yield a ∆Ct value. The ∆Ct of normal samples was subtracted from the ∆Ct of the CML samples to yield a ∆∆Ct value, which was converted into relative quantification (RQ) by the formula: 2^−∆∆CT^ [12].

### In silico analysis from public available data repositories

Microarray data from the International Microarray Innovations in Leukemia (MILE) study group was analyzed. Expression of *MLL2/KMT2D* (probe 231974_at) and *MLL3/KMT2C* (probe 1557158_at) in 76 CML patients and 73 non-leukemia or healthy bone marrow samples from the MILE study group were obtained through the Bloodspot database [13] in the form of log2 scaled intensity values [14, 15].

### Statistical analyses

Descriptive statistics were used to summarize data. Mann–Whitney test was used for independent non-parametric samples to compare gene expression between CML and control groups and between therapeutic response groups. The non-parametric Kruskal–Wallis test, followed by Dunn’s Multiple Comparison test, was used to compare gene expression among different stages of CML. Chi square goodness of fit test was used to verify if observed proportions differ from the hypothesized ones. The non-parametric Spearman test was performed to check the correlation between *MLL2/KMT2D* and *MLL3/KMT2C* gene expression in each CML patient. Data were analyzed and plots created using the Prism 6 software (GraphPad Software Inc., San Diego, CA, USA). Statistical significance was defined as a one-tailed p value < 0.05 (CI 95%).

## Results

### *MLL2/KMT2D* expression profile in CML patients

**Fig. 1:**
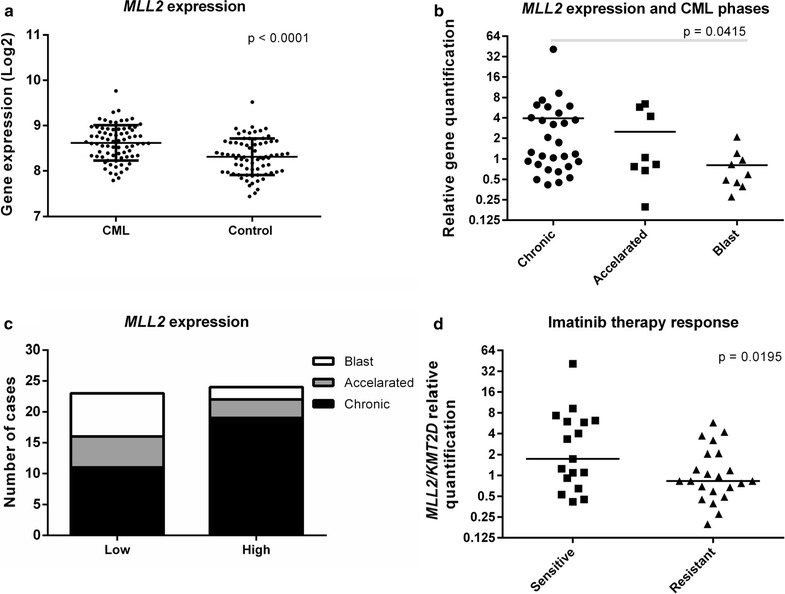
*MLL2/KMT2D* expression profile in CML patients. **a** Data analysis from the MILE study group database revealed that *MLL2/KMT2D* expression is upregulated in 76 CML patients compared to 73 control samples available (microarray probe 231974_at, Mann–Whitney test, p < 0.0001). **b** *MLL2/KMT2D* expression among different phases of CML. The lowest expression level was found in the more aggressive blast phase (29 patients in chronic phase, 8 in accelerated phase and 9 in blast phase, Kruskal–Wallis test, p = 0.0415). **c** The median value of *MLL2/KMT2D* expression was used as the cut-off to dichotomize CML patients in “low” and “high” expression groups. When testing all the combinations of phases, the low expression of *MLL2/KMT2D* was more frequent in the more aggressive blast phase compared to the chronic phase (Fisher Exact one-side test, p = 0.0361). **d** *MLL2/KMT2D* expression in CML patients sensitive or resistant to imatinib therapy. The lowest expression level was found in the patients that did not respond to the therapy (22 resistant and 17 sensitive patients to imatinib, Mann–Whitney test, p = 0.0195)

We initially analyzed gene expression data from the MILE Study in a set of 76 CML patients and 73 non-leukemia or healthy bone marrow samples. Expression of *MLL2/KMT2D* was significantly upregulated in CML patients when compared to control samples (Fig. 1a, Mann–Whitney test, p < 0.0001). Although this difference was evident in at least 2 microarray probes used in the MILE study, in our cohort of 46 patients and 20 healthy donors, we found no significant difference in *MLL2/KMT2D* expression between the groups. However, when we stratified the CML samples according to their different stages, *MLL2/KMT2D* showed a higher expression in chronic phase, with a gradually lower expression in the accelerated phase (twofold) reaching the lowest expression in the aggressive blast phase (fourfold) (Fig. 1b, Kruskal–Wallis test followed by Dunn’s Multiple Comparison test, p = 0.0415). CML patients were subsequently dichotomized in “low” and “high” expression groups, using the median value of *MLL2/KMT2D* expression as the cut-off, and a contingency analysis was performed with the CML phases in these groups. The observed proportions for the low and high expression groups did not differ from the hypothesized proportions (Chi square goodness of fit test). However, when all the combinations of phases were tested with Fisher exact test (2 × 2), low expression of *MLL2/KMT2D* was more frequent among the aggressive blast phase than among the chronic phase (Fig. 1c, Fisher Exact one-side test, p = 0.0361). CML patients were further stratified according to their response to imatinib. Interestingly, *MLL2/KMT2D* expression was significantly reduced in the group of imatinib resistant patients compared to the sensitive ones (Fig. 1d, Mann–Whitney test, p = 0.0195).

### *MLL3/KMT2C* expression profile in CML patients

**Fig. 2:**
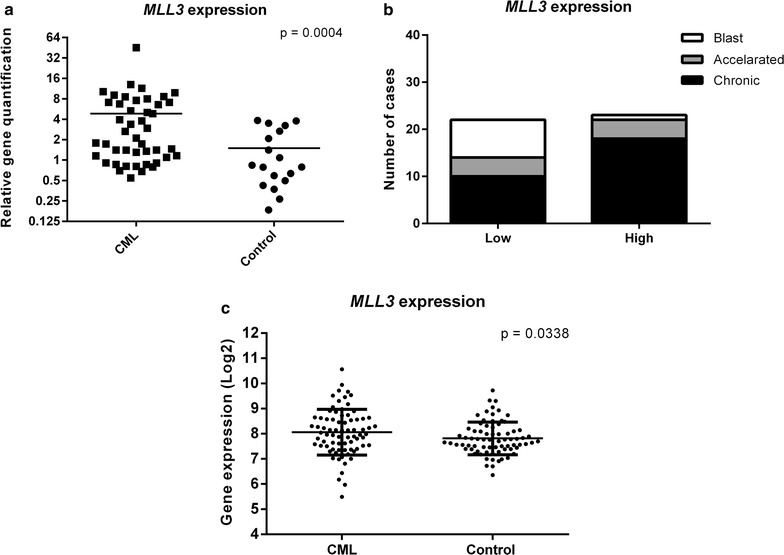
*MLL3/KMT2C* expression profile between CML patients and healthy controls. **a** *MLL3/KMT2C* expression is upregulated in CML patients compared to control samples (45 CML patients and 18 control samples, Mann–Whitney test, p = 0.0004). **b** *MLL3/KMT2C* expression profile frequency among different stages of CML. The median value of *MLL3/KMT2C* expression was used as the cut-off to dichotomize CML patients in “low” and “high” expression groups. The high expression of *MLL3/KMT2C* was more frequent in the chronic phase (Chi square goodness of fit test, p = 0.0212). **c** *MLL3/KMT2C* expression is also upregulated in 76 CML patients compared to 73 control samples from the MILE study group (Microarray probe 1557158_at, Mann–Whitney test, p = 0.0338)

*MLL3/KMT2C* expression was upregulated in CML patients when compared to control samples (Fig. 2a, Mann–Whitney test, p = 0.0004). Considering the different CML phases, *MLL3/KMT2C* did not show significant differential expression among them, although the expression decreases gradually through the accelerated and blast phase. When CML patients were dichotomized in “low” and “high” expression groups and a contingency analysis was performed, as explained above, the observed proportions for the low and high expression groups showed significant difference among the CML phases, with the high expression of *MLL3/KMT2C* being more frequent in the chronic phase (Fig. 2b, Chi square goodness of fit test, p = 0.0212). Finally, when CML patients were stratified according to their response to imatinib, *MLL3/KMT2C* expression did not show any difference between groups.

The difference in *MLL3/KMT2C* expression between CML patients and health controls was also observed in the analysis of the data available from the MILE study group. *MLL3/KMT2C* also showed a higher expression in CML patients when compared to control samples (Fig. 2c, Mann–Whitney test, p = 0.0338).

### Correlation of *MLL2/KMT2D* and *MLL3/KMT2C* expression in CML patients

**Fig. 3:**
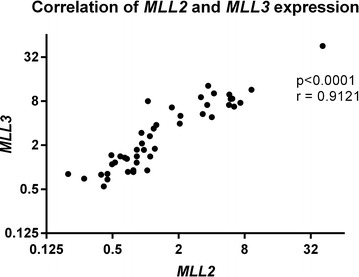
Correlation analysis of *MLL2/KMT2D* and *MLL3/KMT2C*. *MLL2/KMT2D* and *MLL3/KMT2C* gene expression for each CML patient (Spearman Correlation test, p < 0.0001, r = 0.9121, CI 95%)

We investigated whether *MLL2/KMT2D* and *MLL3/KMT2C* mRNA expression levels were correlated in CML samples. We found a strong positive correlation for each CML patient (Fig. 3, Spearman Correlation test, p < 0.0001, r = 0.9121, CI 95%).

### *MLL2/KMT2D* and *MLL3/KMT2C* expression in KCL22 cell lines

**Fig. 4:**
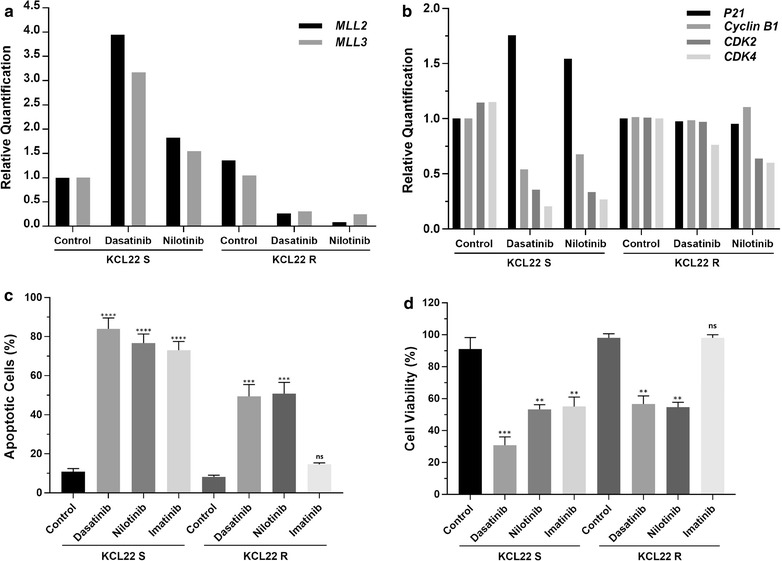
*MLL2/KMT2D* and *MLL3/KMT2C* expression in KCL22 cell lines resistant or sensitive to IM. **a** Expression of both genes was decreased in IM resistant KCL22R cells after treatment with either dasatinib or nilotinib compared to the untreated cells. Treatment with dasatinib or nilotinib in IM sensitive KCL22S cells, resulted in four and threefold increase in the expression of *MLL2/KMT2D* and *MLL3/KMT2C*, respectively. **b** KCL22S cells treated with either dasatinib or nilotinib showed an increase in the expression of *p21* (*CDKN1A*) and a concomitant decrease of *CDK2*, *CDK4* and *Cyclin B1* in comparison to untreated KCL22S control. This difference in the expression was not observed in KCL22R cells after treatment with either TKI compounds. **c** Flow cytometry analysis of annexin-V and propidium iodide (PI) staining of apoptotic cells following dasatinib (10 nM), nilotinib (18 nM) and imatinib (10 μM) treatment or DMSO-treated control in KCL22S and KCL22R, showing percentage of apoptotic cells (annexin-V positive + PI positive + annexin-V and PI double positive cells). (**p < 0.01, ***p < 0.001 and ****p < 0.0001, ns: non-significant). **d** Percentage of viable cells following dasatinib (10 nM) nilotinib (18 nM) and imatinib (10 μM) treatment or DMSO-treated control in KCL22S and KCL22R (**p < 0.01 and ***p < 0.001, ns: non-significant)

In order to further investigate the effects of TKI therapy on the expression of *MLL2/KMT2D* and *MLL3/KMT2C*, we used a pair of KCL22 CML cell lines that are either IM sensitive (KCL22S) or IM resistant (KCL22R). Cell lines were treated with either dasatinib or nilotinib and genes expression levels were subsequently assessed. Expression of both *MLL2/KMT2D* and *MLL3/KMT2C* in KCL22R decreased one to twofold in the treated cells when compared to the untreated control. In contrast, treatment of IM sensitive KCL22S cell line with dasatinib resulted in a four and threefold increase in the expression of *MLL2/KMT2D* and *MLL3/KMT2C* respectively, while treatment with nilotinib slightly increase the expression of both genes compared to the untreated cells (Fig. 4a).

### Association of *MLL2/KMT2D* and *MLL3/KMT2C* with the expression of p53 regulated genes, apoptosis and cell viability in KCL22 cell lines

Due to the previously reported role of *MLL2/KMT2D* and *MLL3/KMT2C* as co-activators of p53 [7, 16, 17] we investigated whether their increased expression after treatment with TKI in KCL22S cells exert any impact in the activation of p53 pathway and apoptosis. KCL22S cells treated with either dasatinib or nilotinib showed an increase in the expression of *p21* (*CDKN1A*) and a concomitant downregulation of *CDK2*, *CDK4* and *Cyclin B1 (CCNB1)* in comparison to untreated KCL22S control, suggestive of p53 activation pathway (Fig. 4b). This difference in the expression was not observed in KCL22R cells after treatment with either TKI compounds. We next examined if there is any correlation between the increased expression of both *MLL* genes after TKI treatment and apoptosis. Interestingly, KCL22S cells, which showed an increase in the expression of both *MLL* genes, had a higher rate of apoptotic cells (p < 0.0001) when compared to KCL22R cells in which expression of the *MLL* genes did not change or was reduced (p < 0.001) (Fig. 4c). Furthermore, apoptotic rate was inversely correlated with cell viability in TKI treated cells (Fig. 4d). As expected, only KCL22R cells treatment with imatinib did not affect apoptosis rate or cell viability.

### Expression of *MLL2/KMT2D and MLL3/KMT2C* in Philadelphia chromosome negative myeloproliferative diseases

In addition to the group of CML patients, we also investigated the expression profile of *MLL2/KMT2D* and *MLL3/KMT2C* genes in other myeloproliferative diseases including polycythemia vera (N = 25), essential thrombocythemia (N = 35) and primary myelofibrosis (N = 20). No significant difference was found in gene expression profile among these chronic myeloproliferative diseases and the normal subjects (N = 18) (data not shown).

## Discussion

MLL2/KMT2D and MLL3/KMT2C subfamily of lysine methyltransferases contains conserved LXXLL motifs that interact directly with nuclear receptor. In collaboration with hormone receptors and transcription factors, this subfamily is the principal responsible for monomethylation of H3K4, especially at transcriptional enhancers regions. Therefore, their reduction, especially in advanced disease stage, could cause abnormalities in enhancers’ activation leading to deregulation of gene expression, which can affect developmental and differentiation programs. In these situations, stem cell state could be maintained, leading to malignant transformation [7, 8, 18, 19]. Although we did not observe a differential expression in the levels of *MLL2/KMT2D* during the indolent chronic phase of our CML group compared to the control group, its expression was clearly reduced during the progression of the disease to accelerated phase and especially to the aggressive blast phase. This reduction was also evident in the group that does not respond to IM therapy. *MLL3/KMT2C* showed a significant upregulated expression in CML patients compared to the control group, which decreased gradually through the accelerated and blast phase, although this differential expression among disease stages was not statistically significant. Interestingly, expression level of *MLL2/KMT2D* and *MLL3/KMT2C* showed a strong positive correlation for each CML patient, suggesting a possible common regulatory mechanism. The analysis of the available data from the MILE study group also showed a heterogeneous expression profile, with *MLL2/KMT2D* and *MLL3/KMT2C* upregulated in the pool of CML patients. This heterogeneity is similar to the one observed in the comparative analysis between the whole pool of CML patients in our cohort and the healthy donors control group. Since the available data from the MILE study group was not stratified according to disease stage, we could not assess the detailed expression profiles of these genes during disease progression.

Previous studies also showed a significantly reduced expression of *MLL2/KMT2D* in breast cancer [11] and a decreased expression of *MLL3/KMT2C* in larynx carcinoma samples when compared to normal adjacent tissue from the same patients, with the lowest expression of *MLL2/KMT2D* and *MLL3/KMT2C* found in the most advanced tumors [10]. Indeed, in recent years, several other studies have been demonstrating that *MLL2/KMT2D* and *MLL3/KMT2C* genes are involved in a multitude of cancers [7, 8]. Inactivating mutations have been identified affecting both genes in several solid tumors, such as breast, esophageal, lung and head and neck carcinomas. However, in many cancers, inactivating mutations can occur in only one of these genes, suggesting that COMPASS-like complexes may act differently according to the cell type. More studies are necessary to clarify the exact mechanisms of these mutated genes in carcinogenesis and if they represent driving events in the malignant transformation process or just later events in disease progression [7, 8]. Moreover, differences in enhancers’ status defined by H3K4me1 profile, which is regulated by MLL2/KMT2D and MLL3/KMT2C, can be found between normal and cancer cells. This differential landscape could improve our understanding of enhancer alterations in carcinogenesis and may constitute a signature that modulate a unique cancer transcriptome [19], as previously shown for colon cancer [20]. A recent work proposed an additional mechanism on how MLL2/KMT2D can contribute to carcinogenesis besides its deregulation of enhancers. It was suggested that *MLL2/KMT2D* inactivation can lead to transcription stress, DNA damage and genome instability, especially in active genes, contributing to cancer evolution and heterogeneity. MLL2/KMT2D seems to be involved in transcript elongation, mediating elongation-associated H3K4 methylation, particularly in histones adjacent to the elongating RNAPII (RNA polymerase II). Therefore, *MLL2/KMT2D* inactivation would lead to replication problems caused by RNAPII undergoing transcription stress, which would originate mutations in these regions [21]. In addition, since MLL2/KMT2D and MLL3/KMT2C are coactivators of the transcription factor p53, being necessary for H3K4 trimethylation and DNA-damage induced expression of p53 target genes, their reduction could lead to all the consequences of decreased activity of p53, including reduced apoptosis and accumulation of DNA damage [7, 16, 17].

Although several tyrosine kinase inhibitors have been improving treatment and survival of CML patients, especially in the initial chronic phase of the disease, still there are no useful markers that can predict genetic evolution, disease progression and development of therapy resistance. The resistant cell line KCL22R was established after treatment with step-wise increasing concentrations of imatinib [22]. This cell line showed no mutation in the *BCR*–*ABL1* gene or increased BCR–ABL protein level. Moreover, Ohmine et al. showed that the level of autophosphorylation of BCR–ABL protein decreased after treatment with imatinib, showing that mechanisms independent of BCR–ABL kinase activity seem to play a role in the acquired resistance to imatinib. They also found that some genes had a differential expression in the resistant cell line, such as RASAP1 and RhoA, important in signal transduction, and C-Myb, a transcription factor with a role in the proliferation of hematopoietic progenitors [23]. A more recent study, used a comparative proteomic approach to identify several proteins differentially expressed in KCL22S and KCL22R that were related to important functional networks, such as cell death, hematological system development and post-translational modification [24]. Although we found no difference in the expression levels of *MLL2/KMT2D* and *MLL3/KMT2C* between KCL22R and KCL22S, we investigated whether treatment of these cell lines with second generation BCR–ABL kinase inhibitors would affect the expression level of both genes. Interestingly, when we treated the IM sensitive KCL22S cell line with dasatinib, we observed a four and threefold increase in the expression of *MLL2/KMT2D* and *MLL3/KMT2C* respectively, comparing to the untreated control. The opposite effect was observed in the IM resistant KCL22R cell line, where expression of both genes was slightly decreased after treatment. A similar trend was found when treating both lines with nilotinib. However, the increase in expression of both genes was not as high as when sensitive cells were treated with dasatinib. Since *MLL2/KMT2D* and *MLL3/KMT2C* were found to be p53 coactivators through their Set1/COMPASS-like complexes [7, 16, 17], we investigated whether the increased expression of both *MLL* genes observed in KCL22S cells after treatment with dasatinib or nilotinib can impact the activation of p53 pathway and apoptosis. Interestingly, we found an associated increase in the expression of *p21* and a concomitant downregulation of *CDK2*, *CDK4* and *Cyclin B1* in KCL22S cells. This expression signature is suggestive of activation of p53 regulated pathways [25–27], associated with a rescue in *MLL3/KMT2C* and *MLL2/KMT2D* expression in response to treatment with dasatinib or nilotinib. In fact, depending on the cellular context, p53 can modulate different tumor suppressor networks leading, for example, to induction of apoptosis, or inhibition of G_1_/S transition through the accumulation of p21, which in turn, inhibit the kinase activity of CDK2 and CDK4 in their cyclin complexes, or leading to the inhibition of G_2_/M transition through the decrease of Cyclin B1 levels [25–27]. In addition, KCL22S cells, which recovered *MLL3/KMT2C* and *MLL2/KMT2D* expression to some degree after treatment with second generation TKI, seem to be more sensitive to apoptosis when compared to the KCL22R cells, where expression of both *MLL* genes was reduced or did not change.

Despite the identification of an association among *MLL3/KMT2C* and *MLL2/KMT2D* increased expression with the transactivation of p53 downstream genes and apoptosis in KCL22S cells, we cannot exclude the possibility that the observed effects on apoptosis are independent of p53 activation. *p53*-induced apoptosis can be independent of its transcriptional function since it still occurs in the presence of protein synthesis inhibitors or in transactivation deficient *p53* mutants [28, 29]. Notwithstanding this link with p53 pathway, the tumor suppressor role of MLL3/KMT2C and specially MLL2/KMT2D complexes seems to be much wider in the cellular context, involving different downstream pathways, such as cAMP signaling [16] which can also vary in a tissue dependent manner.

Although complementary studies with larger cohorts are need, our results suggest that sensitivity to TKI therapy positively affects expression of *MLL3/KMT2C* and specially *MLL2/KMT2D*, further evidencing the potential of these genes as prognostic markers.

## Conclusion

Taken together, our results describe a new correlation between *MLL2/KMT2D* and *MLL3/KMT2C* with CML, showing that the expression of these genes clearly decreased during disease progression. In addition to our findings, future studies are necessary to clarify the role of the members of the conserved COMPASS-like complexes in leukemogenesis. Nevertheless, *MLL2/KMT2D* seems to be associated with disease evolution and may be a potential prognostic marker to predict the development of therapy resistance in CML.

## Abbreviations

CML: chronic myeloid leukemia
TKI: tyrosine kinase inhibitors
IM: imatinib mesylate
Ph chromosome: Philadelphia chromosome
*MLL*: mixed lineage leukemia
FISH: fluorescent in situ hybridization
qPCR: quantitative PCR
Ct: cycle threshold
RQ: relative quantification
MILE: microarray innovations in leukemia
H3K4: histone 3 lysine 4
H3K4me1: histone 3 lysine 4 monomethylation
RNAPII: RNA polymerase II
